# Drain and Air the Land

**Published:** 1875-09

**Authors:** 


### DRAIN AND AIR THE LAND.

The same arguments which we have from time to time applied to vaults and cesspools and outhouses, apply to the drainage of the earth. Free access of atmospheric air to every part of the soil is of the utmost importance. The air assists the various processes of decomposition by which dead animal and vegetable matter is made to yield products of highest value as elements of the food of plants. If the soil is full of water, of course the air cannot get into the soil to perform this office. Hence, drains by drawing off the water beneath, give the air free admission to the soil, and each shower of rain, by displacing the air already present, and then falling through the soil running away in the drains, renews the supply of fresh air. In this way drains are of the greatest benefit. Drains actually diminish the loss of plant food by washing away. Stagnant water is injurious to the roots and plants. They will not grow in it. Draining removes this, and hence the plants send down their roots deeper. Consequently the capability of absorbing nourishment is greatly increased. It is this increased depths of the roots in well-drained soil which render the crops growing on them less liable to suffer from drouth than those on imperfectly drained land.

Although rain, washing the surface and running off by open channels, may and does dissolve and wash away a considerable quantity of nutritive matter, the water which sinks into the land carries these nutritive substances deeper down into the soil and deposits them in the lower portions where the roots of the plants are to be found. Draining causes the rain to pass through a considerable thickness of soil before it runs off, and hence it causes less loss of nutritive matter than is occasioned by rain washing soil as it does in undrained lands, carrying off to the rivers much of the valuable nutritive matter that abounds in the surface.

FOOD FOR HORSES.—I have found great difference of opinion with regard to feeding, and the amount of food necessary for keeping animals, and I resolved to go to headquarters. I spent considerable time in New York, visiting the horse-railroad and the omnibus stables in that city and in Brooklyn, in order to learn their experiences. I found those in charge very courteous. They opened their books and gave me every information desired. To sum up the results, looking over the record of their experience for several years, I found that they had all settled down, each company for itself, as the result of careful and repeated experiments, the details of which I was privileged to observe, upon one uniform rule for horse-railroad horses, and that was twelve pounds of hay and ten pounds of Indian meal per day. In this way, a railroad horse was kept up to his highest condition, and that they were enabled to do their work more satisfactorily than under any other system that has been tried. Oats had been repeatedly used as an article of food, and the cost was carefully compared with that of the Indian meal. It was found at the time, that during the hot weather the feeding of this amount of Indian meal would be injurious; but the result of the experience was, that Indian meal, on the whole, for a railroad or omnibus horse, was the true thing. But they have one very curious practice, the reason of which I am unable to fathom, which I ought to state in connection with this, as possibly bearing upon the subject under discussion. They invariably water all their horses at one o'clock at night. They have an idea, how true it is I do not know, that watering their horses at night adds greatly to their power of digesting food, and prevents injurious consequences.—*J. S. Gould.*

---

Dry paint is removed by dipping a swab with a handle in a strong solution of oxalic acid. It softens at once.

